# Head-to-Head Intra-Individual Comparison of Biodistribution and Tumor Uptake of [^18^F]FAPI-74 with [^18^F]FDG in Patients with PDAC: A Prospective Exploratory Study

**DOI:** 10.3390/cancers15102798

**Published:** 2023-05-17

**Authors:** Emil Novruzov, Frederik L. Giesel, Yuriko Mori, Peter L. Choyke, Mardjan Dabir, Eduards Mamlins, Dominik Schmitt, Christina Antke, Claudio Pinto, Cristian Soza-Ried, Rene Fernandez, Horacio Amaral, Vasko Kramer, Leonardo Badinez

**Affiliations:** 1 Department of Nuclear Medicine, Medical Faculty and University Hospital Duesseldorf, Heinrich-Heine-University Duesseldorf, 40225 Düsseldorf, Germany; frederik.giesel@med.uni-duesseldorf.de (F.L.G.); yuriko.mori@med.uni-duesseldorf.de (Y.M.); mardjan.dabir@med.uni-duesseldorf.de (M.D.); eduards.mamlins@med.uni-duesseldorf.de (E.M.); dominik.schmitt@med.uni-duesseldorf.de (D.S.);; 2 Department of Nuclear Medicine, University Hospital Heidelberg, 69120 Heidelberg, Germany; 3 Molecular Imaging Branch, National Cancer Institute, Bethesda, MD 20814, USA; pchoyke@nih.gov; 4 Departamento Anatomia Patologica, Hospital Sotero del Rio, Santiago 8207257, Chile; 5 Center for Nuclear Medicine and PET/CT Positronmed, Santiago 7501068, Chile; 6 Positronpharma SA, Santiago 7501068, Chile; 7 Instituto Radiooncológico Santiago INRAD, Santiago 7750000, Chile; badinez@yahoo.com

**Keywords:** FAPI, PET, pancreas cancer, fibroblast activation protein, PDAC, FDG, FAPI-74

## Abstract

**Simple Summary:**

The roll-out of the novel pan-cancer tracer family, fibroblast activation protein (FAP) ligands, has opened a new avenue for the diagnosis of numerous epithelial malignancies. These ligands bind to these transmembrane proteins with enzymatic activity, which is predominantly found on the cell membranes of cancer-associated fibroblasts and regulate the interaction of tumor cells and the tumor microenvironment. The upregulation of these membrane proteins enhances tumor growth and leads to poor outcomes. The first studies with Ga-labeled FAP ligands in patients with pancreatic cancer led to very promising results. However, due to production and financial challenges of ^68^Ga-labeling, ^18^F-labeled FAP ligands emerge as the “diagnostic” FAP-tracer with the potential of widespread, economical use in regular healthcare. With this monocentric, prospective study, we aimed to investigate the utility of [^18^F]FAPI-74 PET/CT examination in patients with pancreatic cancer.

**Abstract:**

Background: Radiolabeled fibroblast activation protein (FAP) ligands, a novel class of tracers for PET/CT imaging, have demonstrated very promising results in various oncological, as well as in some benign, diseases with long-term potential to supplant the current pan-cancer agent [^18^F]FDG in some cancer types. Pancreatic ductal carcinoma (PDAC) belongs to the group of epithelial malignancies with a strong so-called “desmoplastic reaction”, leading to a prominent tumor stroma with cancer-associated fibroblasts that exhibit a marked overexpression of fibroblast activation protein (FAP). The first clinical experiences in PDAC with ^68^Ga-labeled FAP ligands suggested superior sensitivity to [^18^F]FDG. However, there is limited data with ^18^F-labeled FAP derivatives, i.e. [^18^F]FAPI-74, yet prospective single- and multicenter trials are already ongoing. In this proof-of-concept study, we sought to evaluate the biodistribution, tumor uptake, and lesion detectability in patients with PDAC using [^18^F]FAPI-74 PET/CT as compared to [^18^F]FDG PET/CT scans for staging. Methods: This study includes 7 patients (median age 69) who underwent both [^18^F]FDG PET/CT with contrast-enhancement and [^18^F]FAPI-74 PET with low-dose CT for primary staging (n = 3) and therapy response control after neoadjuvant (n = 1) or re-staging after palliative therapy (n = 3). The mean interval between PET scans was 11 ± 4 days (range 1–15 days). The [^18^F]FDG and [^18^F]FAPI-74 PET/CT scans were acquired at 64 ± 4.1 min (range 61–91 min) and 66.4 ± 6.3 min (range 60–76 min), respectively, after administration of 200 ± 94 MBq (range 79–318 MBq) and 235 ± 88 MBq (range 90–321 MBq), respectively. Quantification of tracer uptake was determined with SUV_max_ and SUV_mean_. Furthermore, the tumor-to-background ratio (TBR) was derived by dividing the SUV_max_ of tumor lesions by the SUV_max_ of adipose tissue, skeletal muscle, and blood pool. Results: Overall, 32 lesions were detected in 7 patients including primary (n = 7), lung (n = 7), bone (n = 3), lymph node (n = 13), and peritoneal metastases (n = 2). [^18^F]FAPI-74 detected 22% more lesions compared with [^18^F]FDG with a better TBR and visual lesion delineation. In one patient the primary lesion could be detected unequivocally with [^18^F]FAPI-74 but was missed by [^18^F]FDG imaging. Altogether, most of the lesions demonstrated markedly elevated uptake of [^18^F]FAPI-74 with a simultaneous lower uptake in the background, providing a very high visual contrast. Conclusion: To the best of our knowledge, this is the first, prospective, intra-individual investigation comparing [^18^F]FAPI-74 with [^18^F]FDG imaging in PDAC with encouraging results. These pivotalresults supporta larger, multicentric, prospective study to determine the value of [^18^F]FAPI-74 in detecting and staging PDAC in comparison with current standard of care imaging.

## 1. Introduction

Pancreatic ductal adenocarcinoma (PDAC) accounts for 95% of all pancreatic neoplasms and represents one of the most lethal malignancies. PDAC often has an insidious course leading to delayed initial diagnosis, advanced disease at the time of diagnosis, and a relatively rapid downhill course. The 5-year survival rate is less than 10% and, after treatment, recurrence rates of up to 80% have been reported [1,2]. A characteristic feature of PDAC is a very prominent, dense, fibrotic tumor stroma formed by an abundant extracellular matrix and stromal cells, including cancer-associated fibroblasts (CAFs) and immune cells that encompass up to 80% of the tumor mass. The resulting high interstitial pressure restricts tumor vascularization and leads to a hypoxic tumor environment and nutrient deprivation which selects for aggressive tumors exhibiting proliferation, invasiveness, metastasis, and therapy resistance. Although early PDACs are not highly metabolically active, at some point a “metabolic switch” results in intense glucose uptake by cancer and stromal cells [3,4].

**Figure 1:**
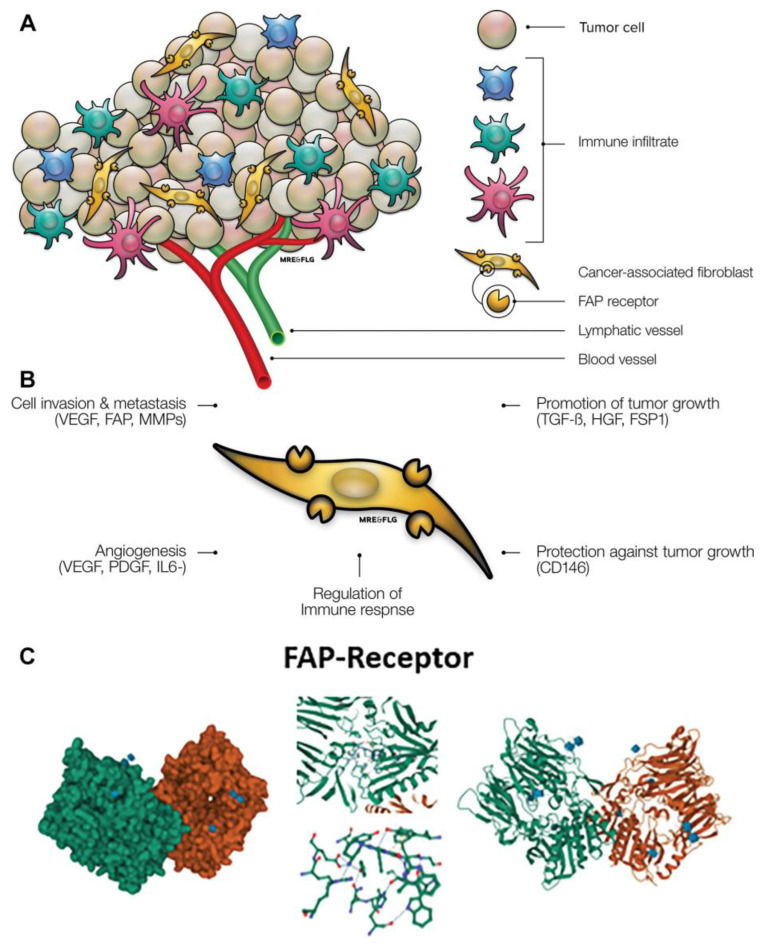
(**A**) Tumor and tumor stroma interaction. (**B**) Various functions of cancer-associated fibroblasts through different transmitters and pathways in tumor stroma. (**C**) Gaussian surface (left) and fibroblast activation protein (FAP-α; PDB: 1Z68) (right) [6]. The illustrations in the middle show the interaction of relevant amino acids within FAP with its ligand linagliptin. FSP1 = fibroblast-specific protein, HGF1 = hepatocyte growth factor, IL6 = interleukin 6, MMP = matrix metalloproteases, PDGF = platelet-derived growth factor, TGF-β = transforming growth factor β, and VEGF = vascular endothelial growth factor (adapted from [7]).

CAFs arise mostly from pancreatic stellate cells as well as mesenchymal stem cells (MSCs), bone marrow-derived stem cells, and/or endothelial cells. CAFs represent activated fibroblastic cells in the tumor microenvironment (TME) that differ from normal quiescent fibroblasts in their phenotype, function, and location. CAFs promote malignant transformation and tumor proliferation by the expression of cell surface markers such as surface-like alpha-smooth muscle actin (α-SMA), fibroblast activation protein (FAP), vimentin, fibroblast-specific protein 1 (FSP1), podoplanin (PDPN/gp38), and platelet-derived growth factor receptor alpha and/or beta (PDGFRα/β) and several chemokines, cytokines, growth factors, miRNAs, exosomes, and metabolites (Figure 1) [5].

Among these targets, FAP appears to be the most relevant cell-surface biomarker. It is a type II membrane-bound glycoprotein with dipeptidyl peptidase and endopeptidase activity. FAP is overexpressed particularly in epithelial malignancies exhibiting rapid tumor growth and proliferation, and thus expression is linked to a poor prognosis. In the case of PDAC, FAP is not only overexpressed by CAFs, but also by the cancer cells themselves, indicating a potential autocrine loop between CAFs and cancer cells [6,8]. Due to its role in tissue remodeling and expression on activated fibroblasts, FAP overexpression is also observed in several non-oncologic, fibrotic diseases including liver cirrhosis, pancreatitis, or idiopathic pulmonary fibrosis [9]. 

Standard-of-care imaging modalities such as contrast-enhanced computed tomography (ceCT), MRI, or ultrasonography are limited in their ability to detect primary tumors and their metastases, and are also limited in therapy response monitoring. [^18^F]FDG PET/CT scans with contrast enhancement have been shown to outperform ceCT alone; however, this combined modality approach is not routinely recommended in guidelines. This is due to the high cost of the exam, its low specificity for cancer, and poor image contrast due to high physiological glucose consumption of normal tissue and frequent confounding inflammatory changes in the pancreatitis [10,11].

Consequently, this unmet clinical need has led to the consideration of FAP-targeted PET agents, which appeared to outperform [^18^F]FDG in several epithelial malignancies including the PDACs [12,13,14]. Pioneering studies by Giesel et al., Röhrich et al., Liermann et al., and Mona et al. in patients with PDAC demonstrated the improved efficacy of ^68^Ga-labeled FAP ligands over both ceCT or [^18^F]FDG PET/CT examinations [15,16,17,18,19]. However, there are several challenges to the ^68^Ga-labeling of radiopharmaceuticals including high positron energy of ^68^Ga with a resulting poor spatial resolution, limited batch size due to limits in the output of ^68^Ge/^68^Ga generators, and the short nuclide half-life (67.7 min), which mandates local production rather than centralized production. In addition, the large positron range of ^68^Ga leads to poorer imaging. The introduction of ^18^F-labeled FAPI tracers with a longer nuclide half-life (109.7 min) and the potential for large batch sizes opened a promising new avenue for cost-effective, large-scale tracer production with widespread use and probably even more favorable tracer characteristics than ^68^Ga-labeled FAPI tracers [20,21]. 

Here, we report on a prospective, exploratory, proof-of-concept study in which we compared the biodistribution, tumor uptake, and lesion detectability of PDAC using [^18^F]FAPI-74 and a contrast-enhanced [^18^F]FDG PET/CT scan.

## 2. Materials and Methods

### 2.1. Clinical Study Design and Patient Cohort

We conducted a prospective exploratory translational study of [^18^F]FAPI-74 imaging in comparison with [^18^F]FDG PET/CT imaging in a cohort of 7 patients between May 2021 and February 2023. The patients were all under treatment for biopsy-proven PDAC, and were enrolled in the study according to clinical standard-of-care indications (approved by the regional ethics committee board (CEC SSM Oriente/19062020)). The conduct of the study was carried out in accordance with the Declaration of Helsinki, Good Clinical Practices, and national regulations. Oral and written informed consent were obtained from all patients on an individual-patient basis following national regulations.

### 2.2. Radiochemistry and Image Acquisition

[^18^F]FAPI-74 was produced in accordance with local GMP regulations using a procedure modified from [20]. Patients were instructed to fast for at least 4 h before [^18^F]FDG PET/CT, and a normal blood glucose level in the peripheral blood was ensured for the [^18^F]FDG PET/CT scan, while for the [^18^F]FAPI-74 PET/CT scan no specific preparation was required. All participants underwent both [^18^F]FAPI-74 and [^18^F]FDG PET/CT whole-body scans on the same scanner within a time interval of 11 ± 4 days (range 1–15 days) and without any change in treatment during the interval. The PET/CT scans were performed at two different PET/CT scanners with interchangeable acquisition protocols using an emission time of 2–4 min per bed position, depending on patient body weight (3 patients on Biograph Vision and 4 patients on Biograph Flow mCT20; both from Siemens, Erlangen, Germany) (Supplementary Materials, Table S1). The [^18^F]FDG scans were performed using contrast-enhanced CT followed by [^18^F]FAPI-74 PET/CT scans using low-dose CT for anatomical localization and attenuation correction. The median injected activity for [^18^F]FAPI-74 and [^18^F]FDG PET/CT was 245 MBq (range, 90–321 MBq) and 239 MBq (range, 79–318 MBq) and the median uptake time was 68 min (range, 60–76 min) and 64 min (range, 61–91 min), respectively. Vital signs were monitored for all patients before and at the end of the intervention and no adverse events or drug reactions were observed.

### 2.3. Image Analysis

Tracer uptake in lesions was quantified by mean and maximum standardized uptake values (SUV_mean_ and SUV_max_). Tumor-to-background ratio (TBR) was derived by dividing the SUV_max_ of tumor lesions by the SUV_max_ of adipose tissue and skeletal muscle in the gluteal region and blood pool in the descending aorta. Circular regions of interest (ROI) were placed over the normal organs by one UKD investigator (EN; supervised by FLG). For small organs (thyroid, parotid gland, myocardium, oral mucosa, and spinal cord) a diameter of 1 cm was used and for larger organs (brain, muscle, liver, spleen, kidney, fat, aortic lumen, and lung), a larger (2 cm) ROI was used. Each ROI was automatically incorporated into a 3-dimensional volume of interest with a 40% iso-contouring approach using Syngo.via Software (ESoft; Siemens Healthineers, Erlangen, Germany). All lesions with a significant tracer uptake on the [^18^F]FAPI-74 and [^18^F]FDG scans or unequivocally morphological findings on enhanced CT, whether primary or metastatic, were considered to be suitable for further analysis. In the case of patients with disseminated metastases, only the index lesions were considered for further analysis. The lesions with a significant tracer uptake were classified as such when the SUV_max_ were more than two times that of TBR or they were detectable on MIP (maximum intensity projection). All the primary lesions were biopsy-proven and the metastatic lesions were confirmed with further imaging follow-up.

### 2.4. Statistical Analysis

We used descriptive analyses for demographics, tumor characteristics, and tracer uptake. Comparison between [^18^F]FAPI-74 and [^18^F]FDG PET/CT-SUV metrics in tumor and normal tissue as well as TBRs were performed with the paired *t*-test and Wilcoxon–Mann–Whitney Test. A *p* value of <0.05 was considered statistically significant. All statistical analyses were performed using SigmaStat Version 3.5 (Systat Software, Inc., San Jose, CA, USA) and SigmaPlot Version 11.0 (Systat Software, Inc., San Jose, CA, USA) for graphical visualization.

## 3. Results

### 3.1. Patient Cohort

Table 1 depicts the demographic data and previous therapies of all seven patients. The cohort included 6 males and 1 female with a mean age of 70 ± 7 years (range, 57–79). Three patients were receiving palliative chemoradiotherapy due to advanced-stage cancer while 1 patient underwent neoadjuvant chemoradiotherapy. The remaining patients underwent PET/CT examinations as part of the diagnostic work-up in primary staging.

### 3.2. Biodistribution of [^18^F]FAPI-74 in Normal Tissue

**Figure 2:**
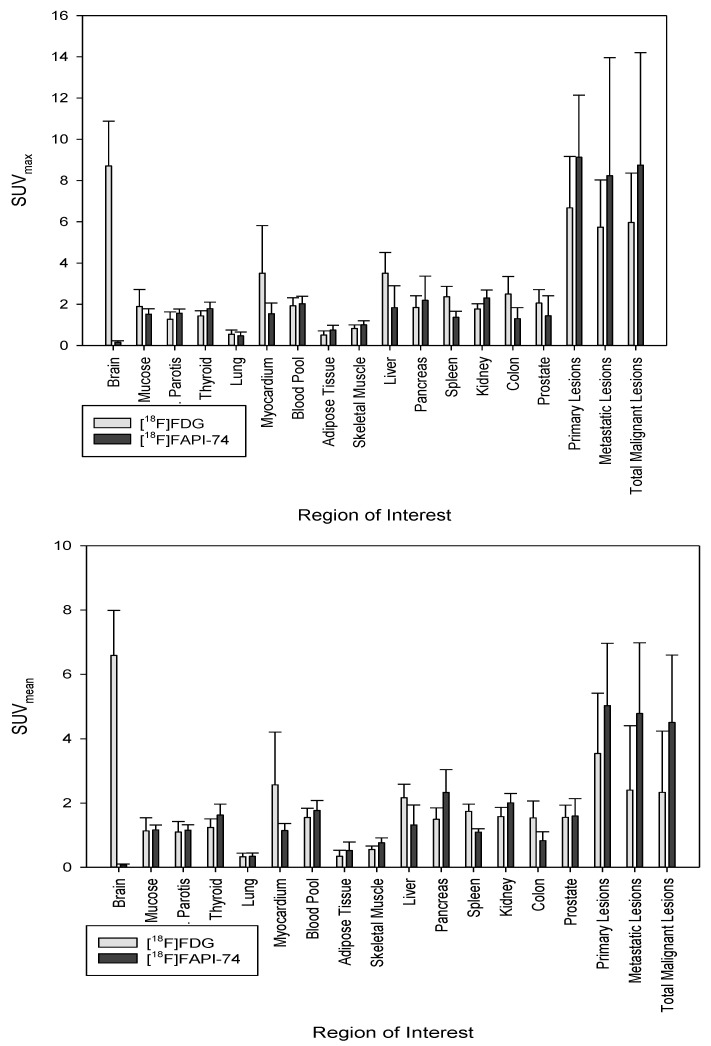
Biodistribution analysis (SUV_max_ and SUV_mean_) of seven patients with PDAC in normal tissue and malignant lesions based on intra-individual comparison of [^18^F]FDG and [^18^F]FAPI-74.

The tumor uptake and biodistribution of [^18^F]FAPI-74 and [^18^F]FDG in normal organs 60 min after intravenous tracer administration are illustrated in Figure 2. The biodistribution of [^18^F]FAPI-74 in normal organs, within likely sites of metastasis, seems to be comparable to that of ^68^Ga-labeled FAP ligands, and thus, much more favorable than that of [^18^F]FDG. However, tracer distribution in background tissue such as blood pool, skeletal muscles, and adipose tissue appears to be comparable with [^18^F]FDG.

### 3.3. [^18^F]FAPI-74 Uptake in Tumor Lesions

**Figure 3:**
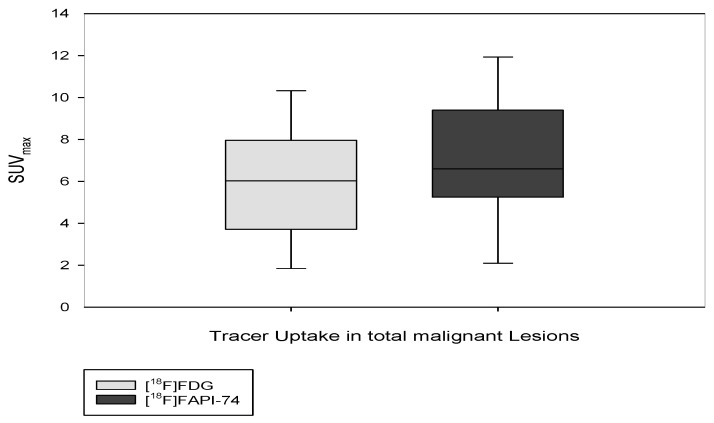
Illustration of tracer uptake (SUV_max_) in overall malignant lesions in a box plot (*p*: 0.61).

**Figure 4:**
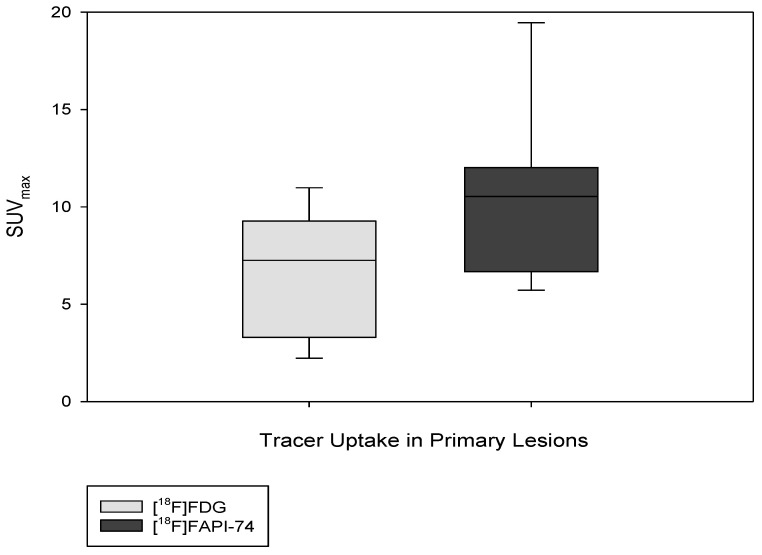
Illustration of tracer uptake (SUV_max_) in primary lesions in a box plot (*p*: 0.01).

**Figure 5:**
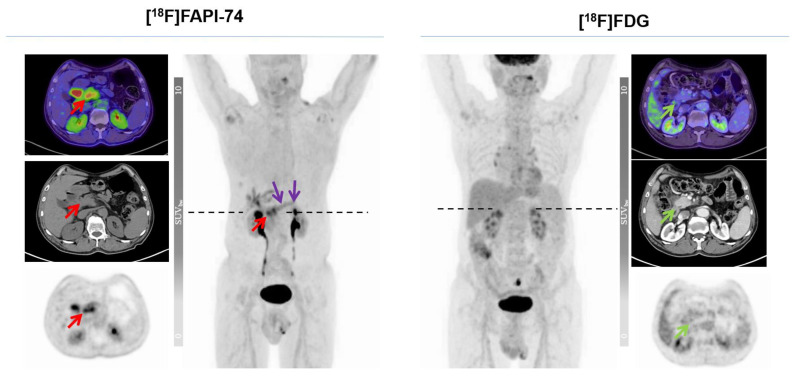
A 69-year-old male patient after neoadjuvant chemoradiotherapy in preoperative therapy response control with no significant uptake in [^18^F]FDG imaging. Red and green arrows indicate the primary lesion, while violet arrows address the confounding, clinically subtle, tumor-associated pancreatitis of the rest of the pancreas (SUV_max_ of the primary lesion in [^18^F]FDG and [^18^F]FAPI-74; 3.3 vs. 5.7, respectively).

**Figure 6:**
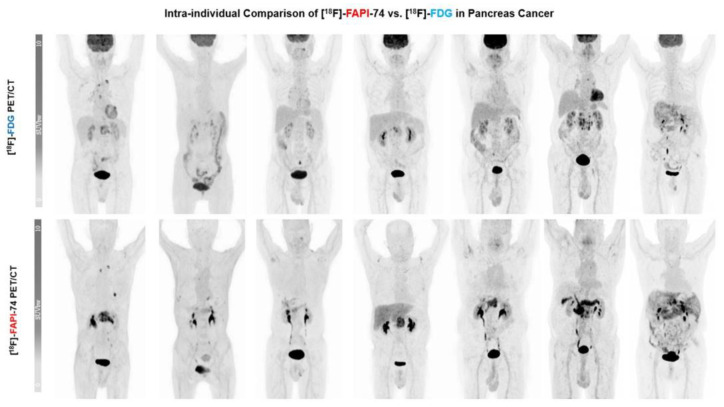
An overview of maximum intensity projections (MIP) in all seven patients, [^18^F]FDG vs. [^18^F]FAPI-74.

The results of [^18^F]FAPI-74 showed that it detected 32 tumor lesions, including all 7 primary lesions and a total of 25 metastatic lesions (lymph node, bone, lung, and peritoneal metastases), whereas the [^18^F]FDG results showed that it detected the primary lesion in only 6 patients and detected a total of 19 metastatic lesions (n  =  25 vs. n  =  32), representing a 22% increased detection rate. Overall tracer uptakes of [^18^F]FAPI-74 and [^18^F]FDG in terms of mean SUV_max_ for all metastatic lesions were 8.2 ± 13.9 and 5.7 ± 2.8 respectively, while the mean SUV_max_ of overall primary tumor lesions were 10.5 ± 4.5 and 6.6 ± 3.2, respectively (Figure 3 and Figure 4). In one patient undergoing neoadjuvant chemoradiotherapy prior to surgery, [^18^F]FAPI-74 detected the primary lesion, which was the only lesion while [^18^F]FDG was negative (Figure 5). Moreover, the other primary lesions exhibited substantially better image contrast and delineation with [^18^F]FAPI-74 (Figure 6). Impressively, [^18^F]FAPI-74 exhibited significantly higher accumulation in primary lesions than [^18^F]FDG (p: 0.01). In contrast, statistical assessment of metastatic lesions revealed no difference in absolute tracer uptake despite subjectively favorable tumor uptake with [^18^F]FAPI-74. Similarly, the tracer uptake assessment with TBR in relation to blood pool, adipose, and skeletal muscle tissue showed no statistically significant difference (Table 2). 

### 3.4. [^18^F]FAPI-74 in PDAC with Confounding Pancreatitis

**Figure 7:**
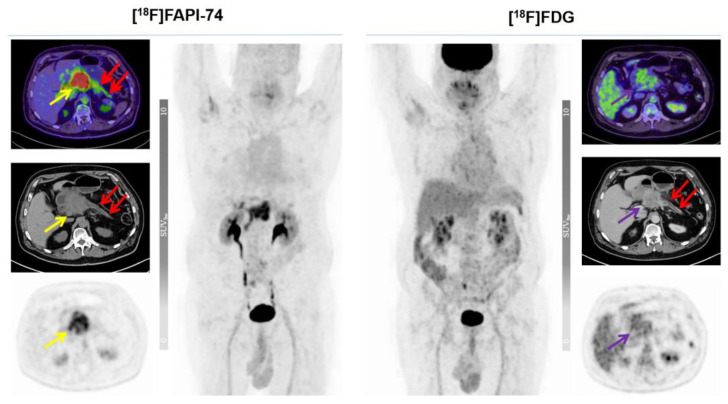
A 66-year-old male patient after palliative chemoradiotherapy with a confounding diffuse pancreatitis and remarkable [^18^F]FAPI-74 uptake in the primary lesion. Yellow and violet arrows indicate the primary lesion, while red arrows address the confounding pancreatitis of the rest of the pancreas (SUV_max_ of the primary lesion in [^18^F]FDG and [^18^F]FAPI-74; 3.4 vs. 10.7, respectively) as in the case depicted in Figure 5.

As described in previous studies, primary lesions in PDAC are frequently accompanied by local, clinically subtle pancreatitis, as these chronic inflammatory changes lead to increased uptake of FAP ligands [9,15,17,18,22]. In this context, we observed increased [^18^F]FAPI-74 uptake in non-tumor pancreas in some patients, but the activity level was lower than that of primary lesions (Figure 5 and Figure 7).

## 4. Discussion

This prospective, exploratory study evaluated the utility of [^18^F]FAPI-74 in PDAC in comparison with contrast-enhanced [^18^F]FDG PET/CT examination. PDAC represents one of the most lethal malignancies with an increasing worldwide incidence and its accurate staging has been a great clinical challenge. Contrast-enhanced CT is the current method of choice but has only a moderate overall diagnostic accuracy; however, it excels in the assessment of the relationship of the tumor to the superior mesenteric artery and vein, and the portal, splenic, and superior mesenteric veins to establish surgical candidacy. Thus, ceCT remains an essential part of the preoperative workup of PDAC. However, post-operative ceCT exhibits limited diagnostic accuracy for local relapse because of numerous overlapping normal tissues [10,11,23,24].

Several studies including meta-analyses and systemic reviews have demonstrated the diagnostic superiority of both the [^18^F]FDG PET scan with low-dose CT and the [^18^F]FDG PET scan with contrast-enhanced diagnostical CT over ceCT alone [11,24]. In particular, the contrast-enhanced [^18^F]FDG PET/CT scan has been shown to be the most accurate and optimal imaging modality because it combines the assessment of surgical operability provided by ceCT with the sensitivity provided by [^18^F]FDG resulting in a sensitivity and specificity of 95% and 81% versus 70% and 80% for ceCT alone. However, contrast-enhanced [^18^F]FDG PET/CT has not been widely recommended in clinical practice guidelines because of its cost and low specificity. Endoscopic ultrasonography (EUS) is also used in the staging of PDAC, but it is much more invasive [24]. Given these limitations, there is pivotal need for improved initial staging and re-staging in tumor recurrence in PDAC imaging.

As pan-cancer imaging agents, the recently introduced FAP ligands have opened a new avenue in the diagnosis of several epithelial malignancies known to overexpress FAP [7,25]. The study of Shi et al. reports an interesting, distinct feature of PDAC regarding FAP expression that it is not only expressed by CAFs but also by tumor cells themselves, which might explain the exceptional uptake of this agent in PDAC [8].

The initial studies with ^68^Ga-labeled FAP ligands in PDAC yielded promising findings resulting in changes in therapy management. For instance, Röhrich et al. compared in a single center study the diagnostic efficacy of ceCT and [^68^Ga]FAPI for PDACs using therapy management change as an endpoint. The use of [^68^Ga]FAPI led to therapy management changes in more than 50% of the patients [17]. Lang et al. showed the role of FAP overexpression, not only in tumor progression and invasiveness but also in predicting malignant transformation of benign pancreas neoplasms to PDAC. They examined 22 patients with intra-ductal papillary mucinous neoplasms (IPMN) known to be prone to malignant transformation. The accurate differentiation of low-grade and high-grade (menacing) IPMN has been a clinical challenge for conventional imaging. The time–activity curves (TAC) acquired during dynamic and later static images of [^68^Ga]FAPI showed increased initial tracer uptake followed by slow washout in high-grade IPMN, whereas the TAC of low-grade IPMN showed lower uptake followed by a rapid washout. This analysis demonstrated an accuracy of more than 80% in predicting high-grade IPMN, which could prevent unnecessary surgery for low-grade IPMN [26].

Nonetheless, ^68^Ga-labeled FAP ligands have some limitations due to the short half-life of ^68^Ga and limited batch production (approximately 2–3 patient doses per elution from the generator) of ^68^Ga. Hence, ^68^Ga-labeled tracers may be a less cost-effective option for nuclear medicine facilities, particularly when considering the increasing financial burden of generators and kits as well as stricter requirements for radiochemistry and infrastructure. Therefore, nuclear medicine facilities without appropriate in-house cyclotrone and clean room labeling would most probably opt for ^18^F-labeled FAP ligands. In addition, the high positron energy of ^68^Ga leads to reduced spatial resolution compared with ^18^F radioisotopes, which in turn further limits the detection of smaller, sub-centimetric lesions. On the other hand, radiolabeling of ligands with ^18^F offers numerous advantages such as the possibility of the production of large batches that can be produced centrally and distributed to medical centers and the longer half-life of the radionuclide, which enables transport over long distances to widely distributed nuclear medicine centers. Furthermore, ^18^F-labeled tracers show much better spatial resolution and thus higher detectability of smaller, sub-centimetric lesions due to the lower positron energy of ^18^F (0.65 MeV) vs. ^68^Ga (1.90 MeV).

Given the advantages of ^18^F, further research enabled the development of the NOTA-conjugated FAP-derivative FAPI-74, which exhibits rapid uptake and clearance. [^18^F]FAPI-74 exhibits a slightly shorter tumor retention time than ^68^Ga-labeled theranostic FAPI agents, but the differences are not significant [20]. Recent investigation demonstrated the feasibility of [^18^F]FAPI-74 with promising results in staging and re-staging of lung cancer patients [27]. Moreover, alongside with further studies, [^18^F]FAPI-74 has been suggested to be interchangeable with other ^68^Ga-labeled FAP ligands in terms of biodistribution in normal organs and tumor uptake [27,28].

The current study revealed a number of interesting comparisons between [^18^F]FAPI-74 and [^18^F]FDG in primary and metastatic PDAC lesions. The evaluation of [^18^F]FAPI-74 biodistribution appeared to be more favorable than [^18^F]FDG with a higher tracer uptake both in primary and metastatic lesions, whereas normal tissues had negligible background activity (Figure 2). Due to this favorable tracer distribution, [^18^F]FAPI-74 outperformed [^18^F]FDG imaging by detecting about 22% more malignant lesions. The [^18^F]FAPI-74 imaging detected the primary lesions with high tracer uptake in all seven patients, whereas [^18^F]FDG imaging detected only six primary lesions due to reduced uptake and beyond that with poorer tumor delineation. Furthermore, [^18^F]FAPI-74 showed superior uptake in primary lesions compared with [^18^F]FDG (*p* < 0.05). Although the visual and numerical detectability of metastatic lesions was better in [^18^F]FAPI-74 imaging, statistical evaluation of specific tracer uptake of various malignant lesions revealed no significant difference between both tracers. In addition, TBRs were comparable between these tracers in terms of blood pool, adipose tissue, and skeletal muscle, as [^18^F]FAPI-74 exhibits higher background uptake than other ^68^Ga-labeled FAP ligands and thus was comparable to [^18^F]FDG. However, regarding the biodistribution in metastasis-target organs, [^18^F]FAPI-74 exhibits a comparable uptake to other ^68^Ga-labeled FAP ligands, but more favorable uptake than [^18^F]FDG.

Local pancreatitis near the primary PDAC was identified in three patients and could be distinguished from cancer by its lower and more diffuse uptake. The increased tracer uptake was only observed in FAP imaging and there was no intervention or therapy between the intra-individual scans. Thus, we identified these changes as clinically subtle, tumor-associated pancreatitis which was also reported to be a classical pitfall in FAP imaging in previous studies [9,15,18,29,30]. The primary lesions were still clearly delineated by [^18^F]FAPI-74 uptake from the pancreas despite the increased background; therefore, a subsequent dual-time measurement was not necessary.

In view of the promising imaging results, FAP-directed radioligand therapy draws increasing attention. However, the first studies with ^153^Sm-, ^90^Y-, ^188^Re-, and ^177^Lu-labeled FAP ligands delivered very heterogenous results due to some inherent shortcomings of current FAP ligands such as short tumor retention time and only indirect effect on tumor cells, namely, over CAFs. Thus, the goal of the ongoing research is to prolong the tumor retention time of FAP ligands and optimally combine the radioligand therapy with other therapy modalities such as immunotherapy [31,32].

There are several limitations to this study. The small size of the cohort does not allow us to draw any definite conclusions on the diagnostic value of [^18^F]FAPI-74. Nevertheless, the initial results of tracer biodistribution and uptake in malignant lesions seem to indicate that [^18^F]FAPI-74 is preferred to [^18^F]FDG imaging. Larger studies will be needed to confirm this initial impression.

## 5. Conclusions

To the best of our knowledge, this is the first prospective, exploratory study that investigates the utility of [^18^F]FAPI-74 PET/CT imaging in PDAC compared with contrast-enhanced [^18^F]FDG PET/CT. Although the sample size was small, the initial results motivate a large-scale, multicentric, prospective study comparing ^68^Ga-labeled FAP ligands, [^18^F]FAPI-74, and contrast-enhanced [^18^F]FDG PET/CT imaging in the setting of PDACs.

## Figures and Tables

**Table 1:** An overview of clinical data of the patient cohort.

Patient	Age	Sex	Previous Surgery	Previous Chemotherapy	Previous Radiation	Clinical Indication	Additional Findings in [^18^F]FAPI-74	Staging Change
1	57	F	No	No	No	Primary staging	4	No
2	69	M	No	FOLFIRI	No	Therapy response control/primary staging	1	Yes
3	65	M	No	FOLFIRI	Yes	Therapy response control/re-staging	no	No
4	66	M	No	FOLFIRI	Yes	Therapy response control/re-staging	no	No
5	78	M	No	FOLFOX	Yes	Therapy response control/re-staging	no	No
6	79	M	No	No	No	Primary staging	no	No
7	75	M	No	No	No	Primary staging	2	No

**Table 2:** The statistical evaluation of tumor-to-background ratio (TBR) of total malignant lesions revealed no significant difference (*p*  <  0.05).

VOI	Tumor/Blood Pool		Tumor/Skeletal Muscle		Tumor/Fat Tissue	
Tracer	[^18^F]FAPI-74	[^18^F]FDG	[^18^F]FAPI-74	[^18^F]FDG	[^18^F]FAPI-74	[^18^F]FDG
TBR	2.64	2.64	5.24	7.01	9.06	10.13
*p* value	0.14		0.97		0.51	

## Data Availability

The data used and/or analyzed during the current study are available from the corresponding author on reasonable request.

## Supplementary Materials

The following supporting information can be downloaded at: https://www.mdpi.com/article/10.3390/cancers15102798/s1. Table S1: Acquisition Protocol.
