# Supplementary material for: Head-to-Head Intra-Individual Comparison of Biodistribution and Tumor Uptake of [18F]FAPI-74 with [18F]FDG in Patients with PDAC: A Prospective Exploratory Study
Source: Cancers (Basel). 2023 May 17;15(10):2798. doi: 10.3390/cancers15102798 (PMC10216510; doi:10.3390/cancers15102798)
Supplement: Supplementary file 1 [file cancers-15-02798-s001.zip › cancers-2304071-supplementary.pdf]

Supplemental Table S1: Acquisition Protocol

Site	Positronmed, Santiago, Chile	Positronmed, Santiago, Chile
PET/CT scanner	Biograph Vision 450 (6R/64CT), Siemens	Biograph mCT Flow 20-3R
CT reference (mAs)	190	170
CT peak kilovoltage (kV)	100	140
CT slice thickness (mm)	2	2
CT slice increment (mm)	1	2
PET reconstruction	OSEM algorithm (PSF & TOF)	OSEM algorithm (PSF & TOF)
Iterations	4	2
Subsets	5	21
Matrix	220 x 220	200 x 200
Corrections	Gaussian FWHM 4.0 mm	Gaussian FWHM 4.0 mm
